# The CKD bowel health study: understanding the bowel health and gastrointestinal symptom management in patients with chronic kidney disease: a mixed-methods observational longitudinal study (protocol)

**DOI:** 10.1186/s12882-021-02600-x

**Published:** 2021-11-21

**Authors:** Tess E. Cooper, Amy Dalton, Anh Kieu, Martin Howell, Sumedh Jayanti, Rabia Khalid, Wai H. Lim, Nicole Scholes-Robertson, Jonathan C. Craig, Armando Teixeira-Pinto, Michael J. Bourke, Allison Tong, Germaine Wong

**Affiliations:** 1 Cochrane Kidney and Transplant, The Children’s Hospital at Westmead, Westmead, Australia; 2 Sydney School of Public Health, The University of Sydney, Edward Ford Building (A27), Fisher Road, Camperdown, NSW 2006 Australia; 3 Centre for Kidney Research, The Children’s Hospital at Westmead, Westmead, Australia; 4 Westmead Hospital, Westmead, Australia; 5 Sir Charles Gairdner Hospital, Nedlands, Australia; 6 School of Medicine, University of Western Australia, Perth, Australia; 7 College of Medicine and Public Health, Flinders University, Adelaide, Australia; 8 School of Medicine, The University of Sydney, Sydney, Australia

**Keywords:** Chronic kidney disease, Kidney transplant, Dialysis, Gut microbiome, Uremic toxins, Gut microbiota, Gastrointestinal symptoms, Observational longitudinal study, Qualitative interview, Discrete choice experiment

## Abstract

**Background:**

Gastro-intestinal (GI) intolerance is a frequently reported outcome in patients with kidney failure receiving maintenance dialysis and those who have received kidney transplants. Symptoms of GI intolerance (diarrhoea, constipation, bloating, abdominal pain, heart burn, and reflux) are associated with significant reduction in quality of life, morbidity, and increased used of healthcare resources. Having chronic kidney disease (CKD), together with related changes in diet and medication, may alter the gut microbiota and the microbial-derived uraemic metabolites that accumulate in kidney failure, and contribute to various complications including chronic diarrhoea, opportunistic infections, and drug-related colitis. Despite the high disease burden among patients with kidney replacement therapies, GI symptoms are often under-recognised and, consequently limited resources and strategies are devoted to the management of gastrointestinal complications in patients with CKD.

**Methods:**

The CKD Bowel Health Study is a multi-centre mixed-methods observational longitudinal study to better understand the bowel health and GI symptom management in patients with CKD. The program comprises of a longitudinal study that will assess the burden and risk factors of GI intolerance in patients treated with maintenance dialysis; a semi-structured interview study that will describe experiences of GI intolerance (including symptoms, treatment, self-management) in transplant candidates and recipients; and a discrete choice experience to elicit patient preferences regarding their experiences and perspectives of various intervention strategies for the management of GI symptoms after kidney transplantation.

**Discussion:**

This proposed program of work aims to define the burden the GI intolerance in patients with kidney failure and generate evidence on the patients’ experiences of GI intolerance and their perspectives on their clinical and own management strategies of these symptoms, ensuring a patient-centred approach to guide clinical decision making and to inform the best study design for intervention trials.

**Trial registration:**

This study is registered on the Australian New Zealand Clinical Trials Registry (ANZCTR): ACTRN12621000548831. This study has been approved by the Western Sydney Local Health District Human Research Ethics Committee of New South Wales Health (HREC ETH03007). This study is supported by a National Health and Medical Research Council (NHMRC) Australia Investigator Grant (APP1195414), and an NHMRC Australia Postgraduate Scholarship (APP2005244).

## Background

Gastro-intestinal (GI) intolerance is a frequently reported outcome in patients with kidney failure receiving maintenance dialysis and those who have received kidney transplants. Symptoms of GI intolerance (diarrhoea, constipation, bloating, abdominal pain, heart burn, and reflux) are associated with significant reduction in quality of life (in particular anxiety and life participation), substantial morbidity burden, increased used of health care resources, and increased risk of allograft complications (including acute rejection as a result of a reduction in immunosuppression). Prior observational studies reported up to 50% of kidney transplant recipients experience adverse GI side effects from immunosuppression, such as mycophenolate mofetil and tacrolimus. Many patients require dose-reduction of immunosuppressive agents that could predispose to a higher risk of acute rejection [1–6]. Alternative immunosuppressive agents such as enteric-coated mycophenolic acid (EC-MPA, Myfortic®) may be better tolerated, but up to 30% of patients continue to experience symptoms of GI intolerance [7]. Other than dose reduction and/or modification of immunosuppressive regimen, there has been no other proven effective treatment strategies that have been shown to reduce GI intolerance in kidney transplant recipients. In patients treated with maintenance dialysis, the most common symptoms include nausea, vomiting, diarrhoea, constipation, heartburn and reflux. In fact, up to 80% of dialysis patients experience GI intolerance, in particular constipation which is exacerbated by the use of oral phosphate binders [8].

There are many reasons for the observed GI changes and symptoms in kidney disease, but retention of uremic molecules produced by microbial metabolism within the gastrointestinal tract may have played a key role in the clinical syndrome of uraemia and its complications [9]. These microorganisms may be present in higher concentration within the GI tract because of alterations in gut microbiota and colonic flora in patients with advanced stage CKD. In kidney transplant recipients, reduced microbial diversity and reduced relative abundance of commensal bacterial taxa that may be critical in the digestion of complex sugars (e.g., *Ruminococcus, Dorea, Coprococcus,* and *Bacteroides*) may be associated with post-transplant non-infectious diarrhoea [10]. Prior work has also shown that the risk of developing colorectal cancer in patients with CKD is doubled compared to the general population [11, 12]. Factors for the increased risk may include age, male sex, the cumulative dose immunosuppression and disturbances in the gut microbiome [12]. The gut microbiota is not only capable of promoting intestinal homeostasis and antitumor responses but can also contribute to chronic dysregulated inflammation as well as have genotoxic effects that lead to carcinogenesis [13]. Prior work has indicated that microbial dysbiosis, a pathological imbalance in the microbial community, is implicated in the development of colorectal adenomas.

The intestinal microbiota, therefore, represents a new therapeutic target to improve outcomes of CKD including reducing uraemic symptoms and metabolic abnormalities in patients with CKD and kidney failure, delay the progression of CKD, and possibly reduce the development of cancer [10]. Nutritional supplements such as probiotics or synbiotics (the combination of probiotics and prebiotics) which have been shown to modulate the GI ecosystem by reducing harmful effects of bacterial imbalance in the gut, strengthen the immune system, protect the gut barrier, and reduce the detrimental effects of uremic toxins in patients with CKD [12]. As GI disturbances remain a substantial burden for patients with CKD and their caregivers, a greater understanding of how the alteration in microbial diversity contributes to GI intolerance in patients with kidney failure and kidney transplants will inform the most appropriate clinical trial design for novel interventions to address this important patient-centred outcome. This proposed program of work will define the burden of GI intolerance and symptoms in patients with kidney failure on dialysis and with kidney transplants, and understand patients’ experiences of GI intolerance, and their perspectives on the management strategies of these symptoms.

### Research question

To better understand the prevalence, severity and management strategies of GI intolerance in kidney transplant candidates and recipients.

### Objectives

#### Primary objective

To determine the prevalence and risk factors of GI intolerance and symptoms in patients: with kidney failure (dialysis only); before transplant (candidate listed on the transplant waiting list or have a pre-emptive donor); and after kidney transplantation.

#### Secondary objectives

To describe the experiences, symptoms and management strategies of GI intolerance in kidney transplant candidates and transplant recipients.

#### Tertiary objective

To elicit patients’ preferences regarding experiences and perspectives on the management strategies for GI symptoms and intolerance after transplantation.

## Methods

### Study designs

**Fig. 1:**
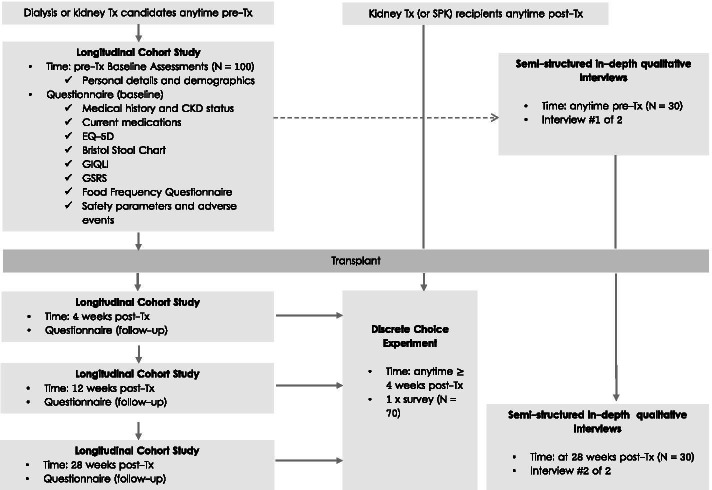
Study Design

An outline of the three study designs and study procedures is displayed in Fig. 1.

1. An observational longitudinal study of the burden and risk factors of GI intolerance in patients treated with kidney replacement therapy
2. A semi-structured interview study in kidney transplant candidates and kidney transplant recipients.
3. A discrete choice experiment of patient preferences regarding the perspectives of the different treatment and intervention strategies for the management of GI symptoms in patients with kidney transplants.

This protocol is developed to address the reporting checklists for:

- STROBE (Strengthening The Reporting of OBservational studies in Epidemiology) for the observational study and discrete choice experience [14];
- COREQ (COnsolidated criteria for REporting Qualitative research) for the qualitative interviews [15].

This study is registered on the Australian New Zealand Clinical Trials Registry (ANZCTR): ACTRN12621000548831. This study has been approved by the Western Sydney Local Health District Human Research Ethics Committee of New South Wales Health (HREC ETH03007).

### Location & Setting

This multi-centre study will be based at two hospitals in Australia: The Western Renal Service (Western Sydney Local Health District and Nepean Blue Mountains Local Health District), New South Wales, Australia (primary location); and the Renal Medicine and Transplantation Department (Northern Metropolitan Health Service), Sir Charles Gairdner Hospital, Western Australia, Australia (secondary location).

### Consent

We will obtain informed consent from each adult participant, caregiver, or legal guardian. There will be no implied consent. To ensure COVID-19-safe practices, we will allow for video or phone verbal consent. Participants can withdraw their consent for the study at any time without affecting their specialist care or disease management in any way.

### Study population

#### Eligibility criteria

To be eligible for this program of work, participants must be: adults aged 18 years and older, with kidney failure (CKD Stage 4 to 5D: estimated glomerular filtration rate = 0 to 29 mL/min/1.73m^2^ and albuminuria > 300 mg/g) [16], on dialysis only, or on dialysis and listed on the deceased donor kidney transplant waiting list, or patients with kidney failure with a planned pre-emptive transplant within 12 months. The study includes both kidney-only and simultaneous pancreas-kidney transplant candidates of any GFR and a minimum one functioning organ. Participants will be recruited into the study irrespective of whether they had current or past experiences of GI intolerance and symptoms.

#### Exclusion criteria

Prior bowel resection, chronic pancreatic insufficiency, or recent infection-related diarrhoea in the last 3 months. Pre-dialysis patients. Transplant recipients we will exclude recent rejection episodes, and unable to give informed consent.

### Recruitment procedure

All eligible patients will be identified using the renal databases and through communication with the medical and nursing staff of the unit. This will be either via phone recruitment or consecutive patients attending the outpatient clinics. Participant consent forms, clearly and succinctly outline the screening procedure and the associated harms will be given to all eligible patients to read through at the face-to-face recruitment by the research staff. Informed consent was written where possible and to ensure COVID-19-safe practices, we will allow for video or phone verbal consent as approved by the ethics committee. For patients with non-English speaking background, a qualified health care interpreter will translate information sheets into the patient’s native language. Participation in the study is entirely voluntary. Patients are in no way obliged to participate and if they do, they can withdraw anytime from the study, with their care unaffected in any way.

#### Longitudinal cohort study

Recruitment of participants will occur anytime pre-transplant and the following will be collected (Table 1):

- General patient contact information, demographic characteristics, medical history, and CKD health status via the Observational Questionnaire (Baseline).
- The baseline assessments, pre-transplant of observational data via the Observational Questionnaire (Baseline) which will collect transplant status, current medications and supplements, general health via the EQ-5D Questionnaire [17], faecal characteristics using the Bristol Stool Chart [18], gastrointestinal symptoms via the Gastrointestinal Symptom Rating Scale (GSRS) [19, 20], quality of life using the Gastrointestinal Quality of Life Scale (GIQLS) [21], personal management strategies of gut health and GI symptoms, dietary patterns using the Food Frequency Questionnaire [22], and safety parameters and adverse events.
- To repeat the instruments, the Observational Questionnaire (Follow-Up) will be administered at 4-, 12-, and 28-weeks post-transplant (28 weeks being the final follow-up time-points). This questionnaire contains the same instruments without the patient contact information and demographic sections.

**Table 1:** Study Visit Schedule

Task	Time points				
	Visit 1		Visit 2	Visit 3	Visit 4
	Recruitment and baseline (anytime pre-Tx)	Tx	4 wk. post-Tx	12 wk. post-Tx	28 wk. post-Tx
**Screening** against eligibility criteria	✓				
**Participant Information Consent Form:** Combined form for all 3 study arms	✓				
**Questionnaire (baseline)**	✓				
**Questionnaire (follow-up)**			✓	✓	✓
**Qualitative Interview**	✓				✓
**Discrete Choice Experiment Survey**				✓	
**Tx:** transplant; **wk:** weeks.					

The primary outcomes are: (1) prevalence of GI intolerance (any severity) in patients with kidney failure and kidney transplants; and (2) change in GI symptom burden before and after transplantation. Secondary outcomes are: (1) risk factors of GI intolerance; (2) stool characteristics; (3) general health status, and (4) quality of life.

Instruments: the observational questionnaires (baseline and follow-up) will collect a broad range of both clinically important and patient-reported outcomes [23] using a variety of validated instruments. The EQ-5D tool is a 5-question Likert scale for measuring general health status according to activities of daily living [17]. The Bristol Stool Chart is a 7-point illustrative scale for identifying different categories of stool characteristics [18]. The GSRS instrument comprises of 15 items combined into five symptom-domains of reflux, abdominal pain, indigestion, diarrhoea and constipation. The GSRS is a graded scale, with a score of 1 representing the absence of any GI symptoms, whereas a score of 7 representing severe GI symptoms [19–21]. The Food Frequency Questionnaire is a validated 131-item dietary assessment tool to capture a patient’s content, frequency, and portion sizes of food and beverages over a specified time period, ranging from 1 to 12 months depending on the question [22].

Statistical analysis: we will undertake a descriptive analysis of the prevalence and management strategies of GI intolerance in patients with kidney failure and have a kidney transplant. Baseline characteristics and dietary patterns will be compared between recipients with and without GI intolerance using chi-square (to compare categorical variables), analysis of variance (compare means for normally distributed variables) and Kruskal-Wallis tests (to compare medians for non-normally distributed variables). Longitudinal mixed models with random intercepts will be conducted to assess the relationship between dietary patterns and GI intolerance, accounting for confounders including age, sex, medication use, and CKD stage.

*Sample size calculation*: If the true prevalence of GI intolerance and symptoms is 7–10% in patients with kidney failure, a sample size of 100 will ensure 95% certainty that the prevalence measure in this sample between 5 to 15%, where n = minimum sample, p = estimated prevalence, q = 100-p, E = 5 (margin of sampling error) $n= pq/{\left(\frac{E}{1.96}\right)}^2$ [24].

#### Semi-structured interview study

##### Participant recruitment and selection

Participants who were identified as eligible for the observational longitudinal study at recruitment (anytime pre-transplant), and who reported the presence of GI symptoms, burden and intolerance, will be invited to participate in the qualitative semi-structured interviews. This will be a longitudinal qualitative study and purposive sampling will be used to ensure a broad range of demographics are included in this study. We plan to enrol a minimum of 30 CKD patients from this study population until saturation. Participants will be interviewed twice: once at baseline (anytime pre-transplant) (Visit 1, Table 1) and once post-transplant at the 28-week follow-up (Visit 4, Table 1).

##### Data collection

A semi-structured interview will be conducted with each participant in their choice of setting either in a clinic room or via video conference. The interviews will be administered by the study research coordinators who are research academics with training in qualitative research. Some participants may be known to the interviewer but only from the recruitment process. The interviewers will not have clinically treated the participants.

The interview guide will include the following sections: (1) demographics; (2) Interview 1: general perspectives and experience of gut health in CKD; perceived causes, impact and management of GI symptoms in CKD (this includes both self-management and medically advised); expectations of transplantation; reasons for participating in the study; (3) Interview 2: experience of being in the study; experience of the transplant; experience of GI symptoms post-transplant.

The guide was developed following a review of the literature and discussion among the research team consisting of multidisciplinary clinicians and researchers and a consumer advisor [25–32].

##### Data analysis

Thematic analysis: interviews will be recorded with the participant consent and transcribed verbatim. Transcripts and field notes will be thematically analysed to independently code all transcripts and notes line by line by two study personnel to identify concepts.

#### Discrete choice experiment

All participants identified as eligible for the observational longitudinal study, as well as post-transplant patients presenting at the renal clinics, will be invited to participate in the discrete choice experiment. We plan to enrol 70 participants and the survey will be administered at either Visit 3 or Visit 4 (Table 1), or anytime within 4 months post-transplant. Participants will be administered the electronic survey directly after attending their usual medical appointment with their surgeon using a clinic computer or tablet.

**Fig. 2:**
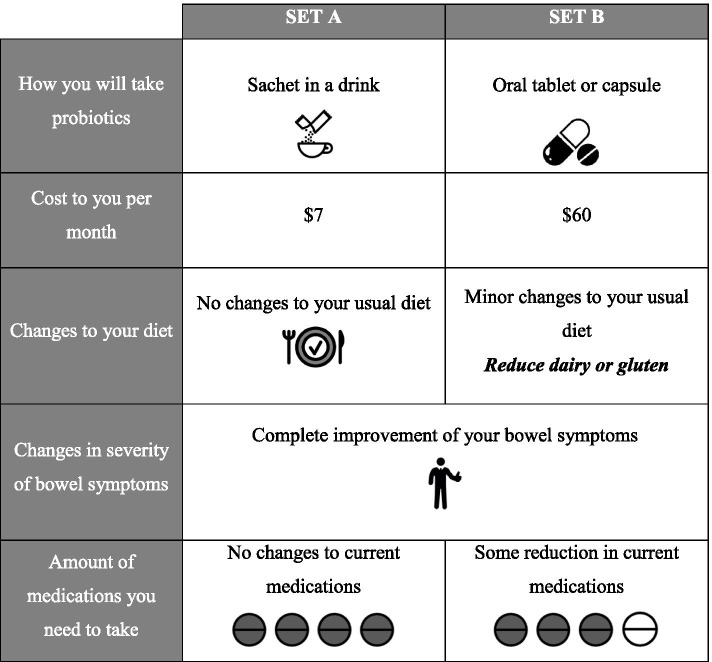
Example of Discrete Choice Analysis questions

Figure 2 provides an example question of the attributes and levels. Participants will be identified by their participant study identification number which will be linked to their demographic information. The survey will be administered as follows: (1) instructions and introduction of the study aim, (2) explanation of attributes, (3) explanation of choice sets. In the survey, participants will be given 5 to 10 choice sets to answer their most preferred scenario of post-transplant treatment options. The results of the first qualitative interview (Study 2) will inform the attributes for this survey. However, the anticipated attributes are: mode of taking supplements; average cost per month; dietary changes; and post-transplant prescribed immunosuppressive medications. The survey was developed following a review of the literature and discussion among the research team consisting of nephrologists and public health (qualitative) academics [33–36].

Statistical Analyses: we will use NLOGIT, version 5.0 [37] to analyse the data. To best the extent to which results were consistent with the researchers’ prior expectations, internal validity will be assessed by examining the signs and significance of parameter estimates [38].

### Privacy/ethical considerations

In regard to written information from questionnaires and electronic survey, all personal information and demographics data will be entered onto a secure web platform. The online database will allow for survey and questionnaire results to be de-identified, using only a study code, to anyone other than the study personnel. The face-to-face qualitative interviews will be identifiable but still linked to each pre-assigned study code. No identifiable information will be shared outside the interview room or video call. For publications and conference presentations, only de-identified outcomes and information will be published in peer-reviewed journals. No identifiable information will be shared during presentations or scientific meetings.

### Storage

All data will be de-identifiable with multiple password-protected entry points and backed-up on secure hospital networks and only approved study personnel will have access to the respective programmes as outlined in the above study methods.

### Retention and destruction of data

Data will be stored for a minimum of 5 years post-publication of the results.

### Potential biases

This research project has been designed to make sure the researchers interpret the results in a fair and appropriate way and avoids study doctors or participants jumping to conclusions. We do not believe there are any other potential biases that could arise during the conduct of this study.

## Discussion

GI symptoms and intolerance are important and debilitating outcomes for patients with kidney failure and transplants. Understanding the risk factors can inform patients, clinicians and other health professionals about the potential treatment strategies, stratification of an individual’s risk of the adverse outcomes in the development of care, policy planning and to provide the necessary evidence that underpins future intervention trials. Interventions such as probiotics or synbiotics could possibly reduce the harmful effects of bacterial imbalance, strengthen the immune system, protect the gut barrier, and reduce the detrimental effects of uremic toxins in patients with CKD.

## Data Availability

Not applicable.

## Abbreviations

CKD: Chronic kidney disease
EC-MPA: Enteric-coated mycophenolate acid
EQ-5D: EuroQol 5-Dimenson (scale)
GI: Gastrointestinal
GIQLI: Gastrointestinal quality of life index
GSRS: Gastrointestinal symptom rating scale
NHMRC: National Health and Medical Research Council
